# European Surveillance Network for Influenza in Pigs: Surveillance Programs, Diagnostic Tools and Swine Influenza Virus Subtypes Identified in 14 European Countries from 2010 to 2013

**DOI:** 10.1371/journal.pone.0115815

**Published:** 2014-12-26

**Authors:** Gaëlle Simon, Lars E. Larsen, Ralf Dürrwald, Emanuela Foni, Timm Harder, Kristien Van Reeth, Iwona Markowska-Daniel, Scott M. Reid, Adam Dan, Jaime Maldonado, Anita Huovilainen, Charalambos Billinis, Irit Davidson, Montserrat Agüero, Thaïs Vila, Séverine Hervé, Solvej Østergaard Breum, Chiara Chiapponi, Kinga Urbaniak, Constantinos S. Kyriakis, ESNIP3 consortium, Ian H. Brown, Willie Loeffen

**Affiliations:** 1 Anses, Ploufragan-Plouzané Laboratory, Swine Virology Immunology Unit, Ploufragan, France; 2 Technical University of Denmark, National Veterinary Institute, Copenhagen, Denmark; 3 IDT Biologika GmbH, Dessau-Rosslau, Germany; 4 Istituto Zooprofilattico Sperimentale della Lombardia e dell'Emilia Romagna, Parma, Italy; 5 Friedrich-Loeffler-Institute, Greifswald-Insel Reims, Germany; 6 Ghent University, Faculty of Veterinary Medicine, Laboratory of Virology, Merelbeke, Belgium; 7 National Veterinary Research Institute, Pulawy, Poland; 8 Animal Health and Veterinary Laboratories Agency, Weybridge, Surrey, United Kingdom; 9 National Food Chain Safety Office, Veterinary Diagnostic Directorate, Laboratory for Molecular Biology, Budapest, Hungary; 10 Hipra, Gerona, Spain; 11 Finnish Food Safety Authority, Veterinary Virology, Helsinki, Finland; 12 University of Thessaly, Faculty of Veterinary Medicine, Laboratory of Microbiology and Parasitology, Karditsa, Greece; 13 Kimron Veterinary Institute, Rishon L'Tzion, Israel; 14 Laboratorio Central Veterinario-Sanidad Animal, Algete, Spain; 15 Merial, Lyon, France; 16 Central Veterinary Institute of Wageningen UR, Lelystad, The Netherlands; Duke-NUS Gradute Medical School, Singapore

## Abstract

Swine influenza causes concern for global veterinary and public health officials. In continuing two previous networks that initiated the surveillance of swine influenza viruses (SIVs) circulating in European pigs between 2001 and 2008, a third European Surveillance Network for Influenza in Pigs (ESNIP3, 2010–2013) aimed to expand widely the knowledge of the epidemiology of European SIVs. ESNIP3 stimulated programs of harmonized SIV surveillance in European countries and supported the coordination of appropriate diagnostic tools and subtyping methods. Thus, an extensive virological monitoring, mainly conducted through passive surveillance programs, resulted in the examination of more than 9 000 herds in 17 countries. Influenza A viruses were detected in 31% of herds examined from which 1887 viruses were preliminary characterized. The dominating subtypes were the three European enzootic SIVs: avian-like swine H1N1 (53.6%), human-like reassortant swine H1N2 (13%) and human-like reassortant swine H3N2 (9.1%), as well as pandemic A/H1N1 2009 (H1N1pdm) virus (10.3%). Viruses from these four lineages co-circulated in several countries but with very different relative levels of incidence. For instance, the H3N2 subtype was not detected at all in some geographic areas whereas it was still prevalent in other parts of Europe. Interestingly, H3N2-free areas were those that exhibited highest frequencies of circulating H1N2 viruses. H1N1pdm viruses were isolated at an increasing incidence in some countries from 2010 to 2013, indicating that this subtype has become established in the European pig population. Finally, 13.9% of the viruses represented reassortants between these four lineages, especially between previous enzootic SIVs and H1N1pdm. These novel viruses were detected at the same time in several countries, with increasing prevalence. Some of them might become established in pig herds, causing implications for zoonotic infections.

## Introduction

Pigs play an important role in influenza A virus ecology. They are susceptible to infection with both avian and human influenza viruses, and swine influenza viruses (SIVs) can be transmitted from pigs to other species [1]–[3]. Influenza A viruses contain eight unique segments of single-stranded RNA and are typed according to their surface glycoproteins, the hemagglutinin (HA) and the neuraminidase (NA) [4]. Emergence of a new influenza A virus can occur through several mechanisms: i) interspecies transmission of virus; ii) antigenic change, or drift, in the major viral antigens through mutations; iii) genetic reassortment following exchange of genes between two or more influenza viruses. All three mechanisms have occurred naturally in pigs all over the world, resulting in a complex landscape of different SIV strains circulating in different regions and changing over time. SIVs of H1N1, H3N2 and H1N2 subtypes are enzootic in pigs globally, but their origins as well as their genetic and antigenic characteristics differ between continents and geographic regions [1], [5]. In Europe, the predominant H1N1 SIVs are entirely of avian origin, introduced from waterfowl to pigs in 1979 and referred to as “avian-like swine H1N1” (H1_av_N1) [6]. An H3N2 influenza virus was introduced in European pigs shortly after the human Hong-Kong influenza pandemic in 1968, but only became widespread after it reassorted with the H1_av_N1 virus in the early 1980s, acquiring 6 internal protein genes from the latter lineage [7]–[9]. This “human-like reassortant swine H3N2” (H3N2) is now the dominant genotype of H3N2 virus in European pigs. Finally, another reassortant virus of H1N2 subtype was detected in 1994. This “human-like reassortant swine H1N2” (H1_hu_N2), which is now the predominant virus within this subtype, also exhibits internal genes from the swine H1_av_N1 virus, but acquired the HA gene of a human H1N1 virus from the 1980s [10] and a NA gene of human origin genetically distinct from that of the “human-like reassortant swine H3N2” viruses that emerged 10 years before [11], [12]. Thus, the three major virus lineages share common internal genes, but they have clearly antigenically and/or genetically distinguishable HAs and NAs [12], [13].

These three SIVs have co-circulated for many years within the European pig population even if the prevalence and incidence of individual subtypes varied from one country to another [1], [13]–[15]. New reassortant viruses between these three enzootic SIVs or between SIVs and seasonal human influenza viruses have been detected occasionally since 2000 [1], [12], [13], [16]. However, the situation was rather stable in European pig herds until the emergence in 2009 of the pandemic H1N1 virus (H1N1pdm) of swine origin [17]. H1N1pdm seems to have been generated from an American triple reassortant SIV that acquired the NA and matrix (M) genes from the Eurasian H1_av_N1 lineage [18]–[20]. The H1N1pdm virus was detected in humans several weeks before the first case of reverse zoonosis was reported in a Canadian pig herd [21]. Pigs were shown to be highly susceptible to this swine-origin human virus [22], [23] and the virus caused rapidly numerous outbreaks worldwide, including Europe [24]–[26]. Adaptation of H1N1pdm to swine and ongoing spread in pigs was later demonstrated [27].

Ongoing knowledge of viral strains and lineages circulating in pigs is indispensable for the detection, prevention and control of the disease in pigs, but also to detect novel reassortant viruses that may pose a threat to the human population. From November 2010 to October 2013, the “European Surveillance Network for Influenza in Pigs 3” (ESNIP3) extended and intensified the surveillance programs that were established during two previous European coordination actions, ESNIP1 and ESNIP2 (http://www.esnip3.eu). This paper describes the surveillance programs that were carried out in the different countries involved in ESNIP3, as well as the laboratory tests used for detection, isolation and initial subtyping (HA and NA molecular subtyping and/or virus antigenic subtyping) of SIV strains. It reports the dominant enzootic SIV lineages and emerging reassortant viruses with zoonotic potential that have been identified by ESNIP3 partners in 14 European countries from 2010 to 2013.

## Materials and Methods

### Partners

Within the ESNIP3 consortium, 16 partners were involved in SIV surveillance (Table 1). All partners except Merial SAS contributed directly to both surveillance and laboratory investigations. Merial SAS participated in the network and supported the surveillance under specific collaborations with other partners, especially by enhancing the flow of material from the field to the partner laboratories. FLI, Germany, kindly provided data obtained in a specific research program operational in 2011–2012. Israel was included in this study due to its association with the European Union's Framework Program for Research and Technical Development and its participation in the ESNIP 3 project.

**Table 1:** Influenza surveillance programs carried out by 16 ESNIP3 partners in 17 countries.

Partner*	Country	Passive surveillance	Passive Surveillance											Active surveillance	Serological surveillance
			Involved in surveillance			Area covered		Sampling network				Samples			
			Farmers	Veterinarians	Pathologists	Whole country	Region of country	Voluntary veterinarians	Selected veterinarians	Regional labs	Partner institute	Swabs	Tissue		
AHVLA	United-Kingdom	**x**	x	x	x	x		x		x		x	x		
Anses	France	**x**	x	x	x	x		x		x	x	x	x		**x**
CVI	Netherlands	**x**	x	x	x		x		x	x		x	x		
EVIRA	Finland	**x**	x	x	x	x		x		x		x	x		
FLI	Germany	**x**		x			x	x				x	x		
HIPRA	Spain	**x**		x		x			x			x	x		**x**
IDT	Germany, Netherlands, Poland	**x**		x		x		x				x	x	**x**	**x**
IZLER	Italy	**x**	x	x	x		x	x		x		x	x		
KVI	Israel													**x**	**x**
LCV	Spain	**x**	x	x			x		x			x			
Merial	France, United-Kingdom, Belgium, Italy, Poland, Hungary	**x**		x		x			x			x			**x**
NVRI	Poland, Slovakia, Lithuania, Belarus, Russia	**x**	x	x		x		x			x	x	x		**x**
UGent	Belgium, Netherlands	**x**	x	x	x		x		x	x		x	x		**x**
UTH	Greece	**x**	x	x		x			x			x	x	**x**	**x**
VDD	Hungary	**x**	x	x		x		x				x	x		
VET-DTU	Denmark	**x**		x		x		x				x	x		
Total		**15**	10	15	6	10	5	9	6	6	2	15	13	**3**	**8**

*Full names of partner institutes are given in author's affiliations.

### Inventories of surveillance programs

An inventory of swine influenza surveillance programs implemented in the different participating countries was carried out by sending a questionnaire to partners (S1 Table). This questionnaire asked if the virological surveillance was passive and/or active, if there were serological investigations, who was conducting the different surveillance programs, who paid for sampling and testing, if there were national networks, who provided sampling kits, who reported clinical influenza-like events, who took the biological samples and which areas or regions within the country were monitored.

### Inventory of virological diagnostic methods and diagnostic assistance

An inventory of virological diagnostic methods in use by the partners' laboratories was carried out through a second questionnaire (S2 Table). Partners were asked to indicate their use of molecular tests and/or other protocols to detect influenza A viruses in pig samples, the system (cells, eggs) used for virus isolation, as well as the specific assays run to subtype the viruses detected and/or isolated. The questionnaire also asked partners to list antisera in use for preliminary antigenic subtyping by haemagglutination inhibition (HI) and neuraminidase inhibition (NI) tests.

To assist in virus subtyping by HI and NI assays, a standard panel of five antisera was provided to partners. These sera were produced in specific pathogen free (SPF) pigs at Anses, France, and were preliminarily tested in five laboratories before dispatching. Three of them were hyperimmune sera against strains previously used as reference antigens during ESNIP1 and ESNIP2: A/Sw/Finistere/2889/1982 (H1_av_N1), A/Sw/Flanders/1/1998 (H3N2) and A/Sw/Scotland/410440/1994 (H1_av_N2) [13], [15]. The fourth one was a hyper immune serum raised against a more recent H1_av_N1 SIV isolate, A/Sw/Cotes d'Armor/0388/2009, and the fifth serum was a post-vaccination serum produced in SPF pigs and containing antibodies against A/California/04/09 (H1N1pdm). These hyperimmune and post-vaccination sera exhibited HI titres against homologous viruses that ranged between 1∶640 and 1∶2560.

### Virological surveillance

Data collected and provided by the 15 partners directly involved in virological surveillance consisted of (i) the number of submissions they received (i.e. the number of investigated herds), (ii) the number of successful detections of influenza A viruses (i.e. the number of positive herds), (iii) the number of viruses that were preliminary subtyped (based on HA and/or NA identification), and (iv) the number of viruses identified within each lineage.

## Results

### Inventory of surveillance programs

The inventory of surveillance systems indicated that the mode, extension and intensity of surveillance programs varied between countries and that several partners had reinforced and adapted their surveillance schemes since 2009. All together, the 16 ESNIP3 partners conducted surveillance in 17 different countries (Table 1). All partners except KVI, Israel, used a passive surveillance system based on reporting of clinical acute respiratory disease in pigs.

Outbreaks of acute respiratory disease were reported in general by examining veterinarians, but farmers (or farm technicians) were also involved, directly or indirectly through veterinarians. In six countries, pathologists also contributed to the surveillance. Passive surveillance was carried out effectively either in the whole country or in some administrative regions only, depending on the partner and the type of surveillance program (Table 1). However, even when national programs were implemented, areas with a high density of pig populations remained the most investigated, as for example Brittany in France, northern and central parts of Italy, Northwest Germany and Northeast Spain (http://epp.eurostat.ec.europa.eu/). Specific programs supported by the pig industry and/or vaccine-producing companies contributed to the surveillance on an *ad hoc* basis, for example in France and Germany [28]–[30]. In Finland, France, Denmark and the United Kingdom, the passive surveillance was organized and fully or partly funded at the national level by the government, with voluntary private practitioners liaising with specified veterinary laboratories agreed over identification of SIVs. This procedure designed an algorithm that finally resulted in the submission of positive samples to the ESNIP3 partner laboratory for virus identification [31], [32]. In other countries, partners collaborated with their own network of selected examining veterinarians (Table 1). Most of the time, biological samples were taken by veterinarians or pharmaceutical company's employees, but in several cases samples were also taken by scientists from partner institutes themselves, e.g. when implementing specific research programs in the context of the surveillance.

Acute respiratory disease involving influenza A virus in swine is most often characterized by fever, apathy, anorexia, sneezing, nasal discharge and coughing [2]. Nasal swabs from sick animals were therefore the most common samples (Table 1). In some cases of mortality, tissue samples from the lungs and/or the upper respiratory tract were also taken for virus detection. Clinical materials taken from pigs were sent to local veterinary laboratories and/or the partner laboratory, accompanied with information including at least the location of the farm at the regional level and the date of sample collection.

In some countries, authorities, pig producers and/or research laboratories undertook some active surveillance at the time of the 2009 pandemic for detection of H1N1pdm virus. However, use of active surveillance was restricted to IDT (Germany), KVI (Israel) and UTH (Greece) during ESNIP3 (Table 1). These programs were predominantly structured on an *ad-hoc* basis and for instance were conducted in response to a specific question, *i.e.* about infections with a given virus in a particular region in association with a disease problem. Otherwise they targeted only animals at slaughter.

The main focus of ESNIP3 was virological surveillance so serological surveillance was not routinely carried out by all partners (Table 1). In some cases, serological investigations were undertaken in response to clinical outbreaks and were linked to the passive virological system. Sometimes, serological surveillance was conducted as part of a structured approach which included a selection of farms in a region, aiming to measure seroprevalence and addressing particular questions in relation to the husbandry of pigs and spread of SIVs. During ESNIP3, such serological surveillance programs were implemented by KVI (Israel), UTH (Greece) and NVRI (Poland) in order to provide additional data to the passive surveillance.

### Inventory of diagnostic tests

Nasal swabs and/or lung or trachea tissue samples were processed most often individually, following standard procedures [33].

#### Virus detection and molecular subtyping

The inventory of diagnostic methods indicated that all partners, except one, initially screened clinical samples for detection of any Influenza A virus by carrying out conventional or real-time RT-PCR assays (Table 2). Partners used different protocols either they had developed and/or had adapted from previously published methods (references are given in Table 2). These tests were based on amplification of the matrix (M) gene or more rarely the nucleoprotein (NP) gene. Once influenza A virus was detected in a sample following initial screening by M or NP gene-specific RT-PCR, several laboratories carried out RT-PCR assays specific for HA and/or NA genes for preliminary subtyping (Table 2). Most often, real-time RT-PCR assays that specifically detect H1pdm and N1pdm genes were carried out first. Then, conventional simplex or multiplex RT-PCR assays allowing the amplification of HA and NA genes from European SIVs were applied to H1N1pdm-negative samples by some partners. These multiplex RT-PCRs intended to discriminate either H1 from H3, or H1_av_ from H1_hu_ and H3, or N1 from N2. When combined, these two specific RT-PCR assays allowed users to rapidly identify H1_av_N1, H1_hu_N2 and H3N2 viruses, as well as reassortant viruses that would have exchanged their HA or NA genes, such as rH1_av_N2 or rH1_hu_N2 viruses. In other laboratories, “pan HA” and “pan NA” assays were used to amplify HA and NA genes independent of their lineages which were then identified by partial gene sequencing.

**Table 2:** Overview of RT-PCR techniques and pig sera used by 15 ESNIP3 partners for detection and preliminary subtyping of influenza A viruses in pig samples (rt =  real-time; conv. =  conventional; n.a. =  not applicable; CA =  Cotes d'Armor; Fin = Finistere; Eng =  England; Calif =  California; Scot =  Scotland; Neth =  Netherlands; It = Italy).

Partner	RT-PCR techniques		Sera for routine antigenic subtyping, directed against SIV subtype:			
	for detection	for molecular subtyping	H1_av_N1	H1_hu_N2	H3N2	H1N1pdm
AHVLA	rt, M gene, TaqMan [45]	rt, H1pdm, TaqMan [45]	sw/Eng/195852/92; sw/Eng/604718/96	sw/Scot/410440/94*; sw/Eng/17394/96; sw/Eng/438207/94; sw/Eng/142162/10	sw/Gent/1/84; sw/Eng/163266/87; sw/Eng/201635/92	Calif/7/09
Anses	rt, M gene, TaqMan [47] *commercial kits from LSI and ADIAGENE)*	rt, H1pdm, TaqMan; rt, N1pdm, TaqMan [47] *(commercial kits from LSI and ADIAGENE);* conv., multiplex H1_av_/H1_hu_/H3; conv., multiplex N1/N2 adapted from [64]	sw/Morbihan/0070/05; sw/CA/0388/09*	sw/Scot/410440/94*; sw/CA/0113/06	sw/Flandres/1/98*;	Calif/4/09*; sw/Sarthe/0255/10
CVI	rt, M gene, TaqMan (in-house)	conv., pan HA (+ sequencing); conv., pan NA (+ sequencing) (in-house)	sw/Best/96	sw/Gent/7625/99	sw/Oedenrode/96	Neth/602/09
EVIRA	rt, M gene + H1pdm, TaqMan [45]	conv., pan HA(+ partial sequencing); conv., pan NA (+ partial sequencing) [40]	sw/Best/96	sw/Gent/7625/99	sw/Oedenrode/96	n.a.
FLI	rt, M gene, TaqMan [65]	rt, H1pdm, TaqMan [46]; rt, pan HA; rt, pan NA [29], [66], [67]	n.a.	n.a.	n.a.	n.a.
HIPRA	rt, M gene, SybrGreen [68]	conv., multiplex H1/H3; conv., multiplex N1/N2 [69]	sw/Fin/2899/82*	sw/Scot/410440/94*	sw/Gent/84	n.a.
IDT	conv., M gene [68]	conv., pan H1; conv., H1; conv., H3; conv., N1; conv., N2; [30]	sw/IDT/Re230/93	sw/Visbek/IDT3311/04	sw/IDT/Re220/93	Jena/VI2688/09
IZLER	rt, M gene, TaqMan [22]	conv., multiplex H1_av_/H1_hu_/H3; conv., multiplex N1/N2 [64]	sw/It/1513/98; sw/Fin/2899/82*	sw/It/1521/98; sw/It/2064/99; sw/Scot/410440/94*; sw/It/284922/09	sw/CA/3633/82; sw/It/1523/98; sw/Gent/84	sw/It/290271/09
KVI	rt, M gene, TaqMan [45]	n.a.	sw/CA/0388/09*	sw/Scot/410440/94*	sw/Flandres/1/98*	Calif/4/09*
LCV	rt, M gene, TaqMan [45]	rt, H1pdm, Taq Man [45]; conv., multiplex H1/H3; conv., multiplex N1/N2 [69]	n.a.	n.a.	n.a.	n.a.
NVRI	rt, M gene, TaqMan adapted from [70]; rt, M gene, PriProEt [71]	rt, H1pdm, Taq Man^a^ [45]; conv., multiplex H1_av_/H1_hu_/H3; conv., multiplex N1/N2 adapted from [64] + sequencing [72]	sw/Belgium/1/98	sw/Gent/7625/99	sw/Flanders/1/98	Calif/7/09
UGent	n.a.	n.a.	sw/Belgium/1/98; sw/Fin/2899/82*	sw/Gent/7625/99; sw/Scot/410440/94*	sw/Flanders/1/98*; sw/Gent/84	Calif/7/09
UTH	conv., M gene^a^ [68]	conv., H1pdm^a^ (in-house, adapted from WHO); conv., multiplex H1_av_/H1_hu_/H3; conv., multiplex N1/N2 [64]	sw/Fin/2899/82*; sw/Gent/132/05; sw/CA/0388/09*	sw/Scot/410440/94*; sw/Gent/177/02	sw/Gent/84; sw/Gent/131/05	Calif/7/09
VDD	rt, M gene, TaqMan [45]	rt, H1pdm, TaqMan [45]; conv., pan HA (+ partial sequencing); conv., pan NA (+ partial sequencing) [72], [73]	sw/Belgium/1/98	sw/Gent/7625/99	sw/Flanders/1/98*	n.a.
VET-DTU	rt, M gene, PriProEt [68], [74]; rt, NP gene, SybrGreen [75]	rt, H1pdm (in house); conv., H1; conv., H3; conv., N1; conv., N2 (in-house); rt, pan HA; rt, pan NA [66], [67]	n.a	n.a.	n.a.	n.a.

Virus strains marked with an asterisk (*) were used to produce sera belonging to the ESNIP3 reference panel.

a Since end 2012 (used during the third yearly period of the project).

#### Virus isolation and antigenic subtyping

In case RT-PCR assays for molecular subtyping were not available in the partner laboratory, were not routinely carried out, or were unsuccessful on clinical samples for example due to limited amounts of viral RNA, attempts to isolate the detected virus were initiated either in Madin Darby canine kidney (MDCK) cells or in 10-day-old SPF embryonated chicken's eggs, according to standard procedures [33]. IZSLER, Italy, also used human Caucasian Colon adenocarcinoma (CaCo-2) cells for virus isolation [34].

Once isolated, viruses were submitted to molecular subtyping using the specific RT-PCR assays described above and/or to antigenic subtyping by HI and/or NI tests [33]. Eleven partners performed HI tests using sera either provided by the ESNIP3 reference panel, or produced against local strains, or obtained elsewhere (Table 2).

### Results of virological surveillance

The extensive virological surveillance conducted by ESNIP3 partners from November 2010 to October 2013 included the examination of 9025 herds from 17 countries (Table 3). The intensity of the surveillance programs was highly variable across the countries involved but most often correlated to the level of pork meat production (http://epp.eurostat.ec.europa.eu/). Thus, areas with intensive production inevitably resulted in a greater total number of investigated herds when specific private or public surveillance programs were in place, such as in Germany, Italy, Denmark, France and the United Kingdom. In other countries with high pig population density such as Poland and Spain, visits on farms with acute respiratory syndrome were encouraged by ESNIP3 and increased over time. Thus, an increase of nearly 45% in the number of investigated herds was observed between the first year and the third year of the ESNIP3 program (Table 3). The number of samples collected from a herd was most often comprised between 3 and 15, depending on the surveillance programs, but a herd was considered positive when at least one sample tested positive. These visits on farms resulted in the detection of influenza A virus in 31% of cases (2759 positive herds out 9025). Similar to the numbers of investigations, the frequency of positive cases was highly variable depending on the country, ranging from 3% to 67% (Table 3). However, Influenza A virus infections were everywhere confirmed throughout the year, whatever the season (data not shown).

**Table 3:** Number of investigated and positive herds obtained in each country by the different partners, in the three 12-month periods of the ESNIP3 program as well as in total from November 2010 to October 2013.

Country	Partner	Nov 2010–Oct 2011		Nov 2011–Oct 2012		Nov 2012–Oct 2013		November 2010–October 2013			
		Investigated herds	Positive herds	Investigated herds	Positive herds	Investigated herds	Positive herds	Investigated herds	Positive herds	Percentage positive	Subtyped viruses
Belarus	NVRI	0	0	8	0	12	2	**20**	**2**	**10%**	**0**
Belgium	UGent	36	11	32	9	28	9	**96**	**29**	**30%**	**29**
Denmark	VET-DTU	371	119	320	143	480	226	**1171**	**488**	**42%**	**254**
Finland	EVIRA	14	1	23	0	25	3	**62**	**4**	**6%**	**4**
France	Anses	224	116	296	154	298	163	**818**	**433**	**53%**	**350**
Germany	Total	1094	328	1052	366	1281	405	**3427**	**1099**	**32%**	**874**
	IDT	744	187	1035	352	1281	405	3060	944	31%	754
	FLI	349	140	17	14	0	0	366	154	42%	119
	UGent	1	1	0	0	0	0	1	1	100%	1
Greece	UTH	11	0	17	3	24	6	**52**	**9**	**17%**	**3**
Hungary	VDD	24	7	27	9	51	19	**102**	**35**	**34%**	**38**
Israel	KVI	5	3	0	0	0	0	**5**	**3**	**60%**	**2**
Italy	IZSLER	500	110	556	106	1042	144	**2098**	**360**	**17%**	**179**
Lithuania	NVRI	0	0	0	0	1	0	**1**	**0**	**0%**	**0**
Netherlands	Total	18	7	50	28	19	7	**87**	**42**	**48%**	**39**
	CVI	0	0	26	13	2	1	28	14	50%	11
	UGent	18	7	24	15	4	3	46	25	54%	25
	IDT	0	0	0	0	13	3	13	3	23%	3
Poland	Total	32	15	65	22	88	19	**185**	**56**	**30%**	**30**
	NVRI	32	15	65	22	56	18	153	55	36%	29
	IDT	0	0	0	0	32	1	32	1	3%	1
Russia	NVRI	0	0	0	0	3	2	**3**	**2**	**67%**	**0**
Slovakia	NVRI	1	1	0	0	2	0	**3**	**1**	**33%**	**1**
Spain	Total	59	14	138	24	174	43	**371**	**81**	**22%**	**27**
	HIPRA	54	13	133	24	168	43	355	80	23%	27
	LCV	5	1	5	0	6	0	16	1	6%	0
United-Kingdom	AHVLA	168	39	180	36	176	40	**524**	**115**	**22%**	**57**
**Total**		2557	771	2764	900	3704	1088	**9025**	**2759**	**31%**	**1887**

Percentages of positive herds and total numbers of viruses that were subtyped in the context of the passive surveillance are also given. When several partners were involved in the surveillance in a country, individual numbers are indicated in grey.

Out of the 2759 positive farms, 1887 influenza A viruses (nearly 70% of the total) derived from 14 different countries were HA and NA subtyped, either directly in positive biological samples or after virus isolation (Table 4). Preliminary subtyping showed that in most countries the European enzootic SIVs as well as the H1N1pdm constituted the dominating subtypes (Table 4). Consistent with results obtained within previous ESNIP1 and ESNIP2 actions between 2000 and 2008 [13]–[15], the “avian-like swine H1N1” (H1_av_N1) lineage that emerged in 1979 was the most frequent lineage in every country, reaching 53.6% of frequency in relation to other subtypes identified during the period. By contrast, the enzootic “human-like reassortant swine H3N2” lineage that emerged in 1984 accounted for only 9.1% of the identified viruses. The result was already reported during ESNIP2 that this virus was no longer detected in some regions with high pig population density, whereas it was still prevalent in other parts of Europe. Thus, H3N2 circulated widely in many of the main pig producing regions such as Belgium, The Netherlands, Germany, Italy and Spain but was almost exclusively absent for many years in Denmark, United-Kingdom and France. Only one isolate was obtained in France in early 2012, in the northern part close to the Belgian border [35]. In fact, the enzootic “human-like reassortant swine H1N2” (H1_hu_N2) lineage that emerged in 1994 represented the second most frequent genetic lineage of SIVs in circulation in Europe, being identified in 13% of the viruses characterized in this study. Furthermore, reassortant viruses between the three enzootic SIV subtypes, *i.e*. rH1_hu_N1 and rH1_av_N2, were detected in 7.4% of cases, in several countries. One rH1_av_N2 has been evidenced to have been established in the swine population in Denmark.

**Table 4:** Number of viruses identified within the different haemagglutinin and neuraminidase subtypes and lineages in 14 countries from November 2010 to October 2013.

Country	Number of subtyped viruses	Influenza A virus subtypes and lineages within subtypes						
		H1N1			H3N2	H1N2		Others
		H1_av_N1	rH1_hu_N1	H1N1pdm	H3N2	H1_hu_N2	rH1_av_N2	reass. pdm-like sw HxNy
Belgium	29	16	0	0	10	3	0	0
Denmark	254	68	0	79	0	0	89	18
Finland	4	1	0	3	0	0	0	0
France	350	240	4	7	1	88	10	0
Germany	874	536	6	40	88	94	23	87
Greece	3	0	0	0	3	0	0	0
Hungary	38	19	0	12	4	1	0	2
Israel	2	0	0	1	1	0	0	0
Italy	179	82	2	10	38	39	7	1
Netherlands	39	19	0	0	11	9	0	0
Poland	30	15	0	11	3	1	0	0
Slovakia	1	1	0	0	0	0	0	0
Spain	27	10	0	0	12	5	0	0
United-Kingdom	57	4	0	32	0	6	0	15
**Total**	**1887**	**1011**	**12**	**195**	**171**	**246**	**129**	**123**
**%**		**53.6**	**0.6**	**10.3**	**9.1**	**13.0**	**6.8**	**6.5**

In the period 2010 to 2013, pandemic-like swine H1N1 (H1N1pdm) viruses were identified in numerous countries such as Germany, Denmark, United-Kingdom, Hungary, Poland, Italy, France and Finland (Table 4). In contrast, they were not detected in some other countries having notable numbers of detected influenza cases such as Belgium, The Netherlands and Spain. In Finland, only sporadic detections of the H1N1pdm virus were made several months after the end of the pandemic in humans, but in most countries, this virus was isolated at an increasing incidence over time since 2010 (data not shown). It was also detected in Israel through an active surveillance program, in a pig herd without any clinical signs. Co-circulation of H1N1pdm with European enzootic H1N1, H1N2 and H3N2 SIVs resulted in various reassortment events, leading to the detection of novel reassortant viruses that had mainly exchanged HA and/or NA genes. These viruses accounted for 6.5% of those identified, and therefore at similar levels almost as other reassortant viruses between old enzootic strains. All together, 16.8% of the viruses were H1N1pdm-like viruses or reassortant viruses that had acquired at least the HA or NA gene from the H1N1pdm virus, placing H1N1pdm variants as the second most commonly detected strain after H1_av_N1 viruses.

## Discussion

Following the emergence of H1N1pdm in 2009 and its spread to pigs in many countries worldwide, the OIE and FAO encouraged countries to address and/or reinforce SIV surveillance. The network organized under the auspices of the EU coordinated action ESNIP3 from November 2010 to October 2013, showed that the surveillance systems greatly relied on the involvement of the pig industry, the medical companies and the governments. Thus, at a national level, cooperation between producers, veterinarians, local veterinary laboratories, laboratories conducting reference diagnostic and/or research activities, as well as national authorities, appeared to be a vital part of these voluntary surveillance programs. Amongst the 10 countries with higher intensity pig production (http://epp.eurostat.ec.europa.eu/), differences in numbers of herds investigated during the three year period of the program were probably partly related to the robustness of the local surveillance programs. However, variations in frequency of SIV infections and clinical expression patterns may also depend on different approaches to swine husbandry and disease control. Also, some SIV infections may be sub-clinical, possibly depending on the virus strain and/or environmental conditions, and might be not detected through passive surveillance. Nevertheless, outbreaks of respiratory disease were shown to occur frequently in almost all countries where SIV surveillance was carried out, except in Finland. In this country few outbreaks of respiratory disease with influenza-like clinical signs were investigated despite a surveillance program organized at the national level, suggesting that in Finland respiratory disease is rare.

Harmonisation and standardisation of diagnostic tools for detection and preliminary subtyping of SIVs were proposed within the ESNIP3 program. However, diagnostic capability was highly variable across the continent and appeared to be dependent on resourcing at national level. All but one of the partners participating in surveillance programs deployed RT-PCR assays that were capable of detecting influenza A virus. In a RT-PCR ring trial organized among partners it was demonstrated that all circulating SIV subtypes were correctly detected by participants (data not shown). Also, RT-PCR assays able to specifically discriminate HA and NA genes from the different genetic lineages had been developed and/or adapted in several labs for molecular subtyping in addition to antigenic subtyping. Even if these tests need further development and improvement for greater utility, they have already permitted large-scale screening in biological samples and have led to the rapid identification of many novel reassortant viruses, notably from clinical samples directly, *i.e.* without requiring virus isolation and/or HA and NA sequencing.

Whilst the virological surveillance conducted in this study was based on acute respiratory syndromes, frequencies of herds detected positive for an influenza A virus varied greatly between countries. In most cases, these differences were probably more related to the organization of the surveillance programs themselves than to the SIV prevalence or the efficiency of diagnostic tools. For example, the national surveillance program implemented in the United Kingdom was generally dedicated to any acute respiratory disease of potential viral aetiology with a broad spectrum of clinical signs, whereas in France, the national surveillance network focused more specifically on respiratory disease with influenza-like clinical signs, giving rise to a great difference in the frequency of positive cases (22% versus 57%). However, in Finland, where the number of investigated herds was low despite a national surveillance, percentage of positive herds was also very low (6%), confirming that SIV infections were rare in this country.

As previously described, this ESNIP3 study confirmed that outbreaks of influenza occur throughout the year in pigs, without marked seasonal effect [14], [29]. The surveillance programs provided updated knowledge of the influenza A viruses being maintained within the European pig population since the emergence of the H1N1pdm virus in 2009. They showed that SIVs of H1_av_N1, H3N2 and H1_hu_N2 lineages, which emerged in 1979, 1984 and 1994, respectively, still constitute enzootic lineages in circulation at the European level. Whereas the relative frequencies of the different virus subtypes cannot be assimilated to prevalence values at the country level, they give an indication on the occurrence of the different viruses when they have been calculated from a large enough total number of positive herds and/or large number of investigated herds, also taking into account the size of the pig production. Large differences exist between countries and relative ratio's between these lineages have changed in several countries since the emergence of H1N1pdm, as compared to results of the surveillance previously conducted from 2006 to 2008 [13]. H1_av_N1 viruses, the oldest of the current enzootic European SIVs, were still predominant Europe-wide. They were detected in all countries without exception, irrespective of pig density, either directly through virological investigations or indirectly from complementary serological studies as in those conducted in Greece and Israel (data not shown). However, H1_av_N1 viruses were superseded by H1N2 subtypes in some regions, such as the United Kingdom or Denmark, with respect to incidence, indicating changed dynamics and epidemiology. The proportion of H3N2 viruses identified from 2010 to 2013 was lower than that obtained from 2006 to 2008 [13]. H3N2 viruses were found to be still highly frequent compared to other viruses in several countries but were absent or almost absent from pig populations in others, such as the United Kingdom, France and Denmark. In the United Kingdom and France, H3N2 viruses were responsible for severe outbreaks during several years in the late 80s'-early 90's but were no longer reported since 2000, whilst H1_hu_N2 viruses were becoming more frequent [13]. This raises questions regarding the competitive evolutionary and viral dynamics between strains. Widespread circulation of H1_hu_N2 or, rH1_av_N2 in Denmark, may have led to a decrease in circulation of H3N2, speculatively due to heterosubtypical immunity based on the shared N2 and, again in Denmark, increased fitness of the rH1_av_N2 subtype. However, other countries like Germany, Italy and The Netherlands reported as many H3N2 as H1N2 viruses, suggesting that the disappearance of H3N2 may also rely on other specific herd or regional factors that would require further investigation.

During and after the human pandemic in 2009, the H1N1pdm virus was regularly detected in pigs in many countries, including in Europe, and has since become adapted to the swine [24], [26], [29], [30], [36]–[40]. All these local studies and the ESNIP3 surveillance indicate that the H1N1pdm virus has become well established in the European pig population and now can be considered as a novel enzootic lineage. The lack of detection of any H1N1pdm in some countries such as Spain, Belgium and The Netherlands at the time ESNIP3 was ending could be due to multiple factors. H1N1pdm was easily transmitted from humans to pigs in areas previously free of any other SIVs, as for example in Norway [24] or La Réunion Island [27]. Thus, it could be hypothesized that H1N1pdm did not infect pig populations with a high level of immunity against SIVs and/or highly infected with other SIVs, because of cross-protection and/or viral competition [41]–[43]. Especially in swine-dense areas this high level of immunity can be present, because of frequent circulation of H1_av_N1 in these areas and high usage of vaccination. This may also explain why in France and Germany authentic H1N1pdm virus was preferentially detected in low pig density areas (data not shown). In addition, it cannot be excluded that part of the sporadic infections with H1N1pdm may have been caused by on-going, independent virus transmissions from humans to swine and that these singular transmission events did not lead to sustained holding-to-holding transmission chains. Moreover, infections with H1N1pdm did not induce marked clinical signs sometimes, and that may also account for the difficulty to diagnose them in passive surveillance [27]. In Norway, an active surveillance program conducted in 2009 revealed that only 40% of the H1N1pdm seropositive herds experienced respiratory signs [44]. This potentially raises important questions about the necessity to implement or recommend active virological surveillance in addition to passive surveillance. Finally, it is important to note that H1N1pdm is sometimes difficult to detect using hemagglutination tests after amplification on cells or in eggs. Thus, virus isolation may give false-negative results if only the HI test is used to detect the amplified virus. Therefore partners were advised to establish H1N1pdm specific real-time RT-PCR assays for direct testing of clinical samples, or testing of amplification products, to make sure they were free of H1N1pdm [45]–[47]. Adoption of such methods during ESNIP3 led to the detection of H1N1pdm virus in Poland in 2013, confirming pig infections that were serologically suspected since 2010 in this country [39].

The concomitant circulation of many different virus subtypes in herds increases the risk for co-infections and enables subsequent genetic reassortment. Full genome sequencing initiated on the ESNIP3 virus repository should give greater understanding of genotypes variation in virus detected, but the current study, based on characterisation of HA and NA genes, already identified reassortant viruses involving the four H1_av_N1, H1_hu_N2, H3N2 and H1N1pdm enzootic virus lineages. Moreover, it appeared that the introduction of H1N1pdm into European pig populations led to an increase in the level of incidence of second generation reassortants since 2010. In Germany and Denmark, these “H1N1pdm-like reassortant viruses” mostly consisted of H1N1pdm viruses that acquired an N2 gene from European SIVs (rH1_pdm_N2) [29], [30], [48], [49]. In Germany and Hungary, other reassortants were identified as an H1N1pdm that acquired an N1 gene from the H1_av_N1 lineage [50], [51]. Other various reassortants between H1N1pdm and European SIVs of H3N2 and H1_av_N1 subtypes, including an H3N1 subtype, also have been sporadically detected in pigs in Italy and Germany [30], [52], [53]. In the United Kingdom, additional sequencing led to the identification of H1N2 viruses that acquired internal genes from H1N1pdm [54]. To date, two reassortant viruses of the second generation, *i.e.* the Danish rH1_av_N2 and a German rH1_pdm_N2, have apparently acquired strong transmissibility and therefore were able to maintain themselves in pigs in Denmark and in some German regions, respectively [30], [55]. Other reassortants with genes from the H1N1pdm virus were also detected at an increasing frequency in areas of high pig population density, as for example in the United Kingdom. All these viruses may become enzootic at the European level in the near future and raise further questions as to potential zoonotic agents.

The viruses preliminary subtyped in this study were added to the ESNIP3 virus bank held at AHVLA, Weybridge, United Kingdom. They have been further submitted to full genome sequencing and antigenic characterization that should provide additional understanding on virus evolution, adequacy of diagnostic capabilities, relatedness to currently used vaccines, etc. Especially, sequencing of the internal genes will provide information on reassortment events that could not be detected based on initial HA and NA subtyping. Since 2009 in the United-States, the H1N1pdm was also shown to have frequently reassorted with old enzootic SIVs [56]–[58], and since 2011 clusters of human infection with such a swine-origin influenza H3N2 variant virus (H3N2v) containing the H1N1pdm M gene were reported each year between April and August, at agricultural fair time [59], [60]. Studies conducted in Asia have also demonstrated a massive reassortment between H1N1pdm and asian conventional SIVs, with much of this event taking place at the level of the internal gene constellations [61], [62]. Considering the increasing genetic diversity among SIVs from a given HxNy subtype, running multiplex PCR methods that may allow identification of internal genes in addition to identification of HA and NA encoding genes could be an alternative for determining complete genetic lineages at the preliminary subtyping step, without having to perform full genome sequencing of all viruses, and should be considered in diagnostic algorithms [63].

In conclusion, surveillance of SIV infections on a European scale revealed striking differences between countries participating in the ESNIP3 network regarding the prevalence of ancient and newly established SIV lineages. These differences cannot be explained easily. A careful analysis of trading networks of swine and pork within and between EU member states should give clues as to the likelihood of transboundary SIV transmissions. Continuing the surveillance and providing contemporary updates on the viruses that are currently circulating should enable the detection of novel influenza A virus in the European pig population, once a virus has become established and have the ability to produce disease signs in affected animals with a noticeable herd impact. This provides a useful basis for decisions relating to veterinary health, accounting for better prevention and intervention. Also, a global dissemination of knowledge and information originating from surveillance of influenza A in pigs provides a baseline for the interpretation of the role of swine influenza in zoonotic events and interspecies transmission. This is consistent with pandemic preparedness programs and in line with the “One Health” concept.

## Supporting Information

S1 Table **Questionnaire for the inventory of surveillance programs for swine Influenza in ESNIP3 participating countries.**
(DOCX)

S2 Table **Questionnaire for the inventory of virological diagnostic methods (detection and subtyping) in use by the ESNIP3 partners.**
(DOCX)
