# Supplementary material for: European Surveillance Network for Influenza in Pigs: Surveillance Programs, Diagnostic Tools and Swine Influenza Virus Subtypes Identified in 14 European Countries from 2010 to 2013
Source: PLoS One. 2014 Dec 26;9(12):e115815. doi: 10.1371/journal.pone.0115815 (PMC4277368; doi:10.1371/journal.pone.0115815)
Supplement: S2 Table — Questionnaire for the inventory of virological diagnostic methods (detection and subtyping) in use by the ESNIP3 partners. (DOCX) [file pone.0115815.s002.docx]

**Supporting Information**

**Supporting Information S1**: **Questionnaire for the inventory of surveillance programs for swine Influenza in ESNIP3 participating countries.**

| **Partner** | **Country** | **Type of surveillance*** | | | | **Who is conducting the surveillance programs?** | |
| --- | --- | --- | --- | --- | --- | --- | --- |
|  |  | **Virological** | | | **Serological** |  |  |
|  |  | Passive | Active | |  |  |  |
|  |  |  |  | |  |  | |
| **Passive virological surveillance** | | | | | | | |
| Reporting of clinical influenza-like events | | | | |  | | |
| Sampling | | | | |  | | |
| Investigated areas  (all the country versus specified regions) | | | | |  | | |
| Networks | | | | |  | | |
| Future plans | | | | |  | | |
| **Active virological surveillance** | | | | | | | |
| Networks | | | | |  | | |
| Other studies | | | | |  | | |
| Expected | | | | |  | | |
| **Serological surveillance** | | | | | | | |
| Networks | | | | |  | | |
| Other studies | | | | |  | | |
| Expected | | | | |  | | |

*Enter a cross (X) when existing programs.

**Supporting Information S2**: **Questionnaire for the inventory of virological diagnostic methods (detection and subtyping) in use by the ESNIP3 partners.**

| **Partner:** | | | | | **Country:** | | | | | |  |
| --- | --- | --- | --- | --- | --- | --- | --- | --- | --- | --- | --- |
| **RT-PCRs for Influenza A detection in suitable pig samples** | | | | | | | | | | |  |
| **Target gene** (M, NP…) | **Name of the protocol** | | | **Reference/Source** | | | | **Main characteristics**  (TaqMan, one-step, ready-to-use, multiplex, internal positive control…) | | |  |
|  |  | | |  | | | |  | | |  |
|  |  | | |  | | | |  | | |  |
| **RT-PCRs for subtype specific detection in pig samples and/or for preliminary subtyping after virus isolation** | | | | | | | | | | |  |
| **Target gene** (M, NP…) | **Name of the protocol** | | | **Reference/Source** | | | | **Main characteristics**  (One-step, ready-to-use, multiplex, internal positive control…) | | |  |
|  |  | | |  | | | |  | | |  |
|  |  | | |  | | | |  | | |  |
| **Virus isolation** | | | | | | | | | | |  |
| **System**  (cells, eggs) | | **Cell line/species** | | | | **Reference** | | | **Test for detection of amplified virus**  (HA test, RT-PCR...) | |  |
|  | |  | | | |  | | |  | |  |
|  | |  | | | |  | | |  | |  |
| **Sera for preliminary antigenic subtyping (by HI tests or NI tests)** | | | | | | | | | | |  |
| **Type of serum**  (hyperimmune, post-infection, post-vaccination…) | | | **Virus strain**  (name) | | | | **Subtype and lineage** | | | **Source**  (eg. provided by) | |
|  | | |  | | | |  | | |  | |
|  | | |  | | | |  | | |  | |
